# Pseudophosphorylated αB-Crystallin Is a Nuclear Chaperone Imported into the Nucleus with Help of the SMN Complex

**DOI:** 10.1371/journal.pone.0073489

**Published:** 2013-09-04

**Authors:** John den Engelsman, Chantal van de Schootbrugge, Jeongsik Yong, Ger J. M. Pruijn, Wilbert C. Boelens

**Affiliations:** 1 Department of Biomolecular Chemistry, Institute for Molecules and Materials and Nijmegen Centre for Molecular Life Sciences, Radboud University Nijmegen, Nijmegen, The Netherlands; 2 Department of Biochemistry, Molecular Biology and Biophysics, University of Minnesota Twin Cities, Minneapolis, Minnesota, United States of America; Washington University, United States of America

## Abstract

The human small heat shock protein αB-crystallin (HspB5) is a molecular chaperone which is mainly localized in the cytoplasm. A small fraction can also be found in nuclear speckles, of which the localization is mediated by successional phosphorylation at Ser-59 and Ser-45. αB-crystallin does not contain a canonical nuclear localization signal sequence and the mechanism by which αB-crystallin is imported into the nucleus is not known. Here we show that after heat shock pseudophosphorylated αB-crystallin mutant αB-STD, in which all three phosphorylatable serine residues (Ser-19, Ser-45 and Ser-59) were replaced by negatively charged aspartate residues, is released from the nuclear speckles. This allows αB-crystallin to chaperone proteins in the nucleoplasm, as shown by the ability of αB-STD to restore nuclear firefly luciferase activity after a heat shock. With the help of a yeast two-hybrid screen we found that αB-crystallin can interact with the C-terminal part of Gemin3 and confirmed this interaction by co-immunoprecipitation. Gemin3 is a component of the SMN complex, which is involved in the assembly and nuclear import of U-snRNPs. Knockdown of Gemin3 in an *in situ* nuclear import assay strongly reduced the accumulation of αB-STD in nuclear speckles. Furthermore, depletion of SMN inhibited nuclear import of fluorescently labeled recombinant αB-STD in an *in vitro* nuclear import assay, which could be restored by the addition of purified SMN complex. These results show that the SMN-complex facilitates the accumulation of hyperphosphorylated αB-crystallin in nuclear speckles, thereby creating a chaperone depot enabling a rapid chaperone function in the nucleus in response to stress.

## Introduction

αB-Crystallin is a member of the small heat shock protein (sHSP) family and is regarded as a molecular chaperone with an important role in cellular protection against diverse stress stimuli [1–5]. This is consistent with its high expression in stress-sensitive tissues, like eye lens and muscle [6]. There are numerous examples of its protective function, varying from protecting cytoskeletal components to preventing cell death in general [7,8]. The primary mode of action is to keep partially unfolded substrate proteins in a folding-competent state, allowing quick recovery after stress stimuli [9]. αB-crystallin can be phosphorylated at three different sites: Ser-19, for which the kinase is not known, and Ser-45 and Ser-59, which are phosphorylated by p44/42 mitogen-activated protein kinase and MAP kinase activated protein kinase 2, respectively [10]. Different forms of stress, like heat shock and oxidative ischemic stress, may increase the phosphorylation of αB-crystallin, especially at Ser-59. Ser-19 and Ser-45 are preferentially phosphorylated during the mitotic phase of the cell cycle [5,11]. Phosphorylation of αB-crystallin induces conformational changes which affect the interaction with substrate proteins [12]. The changes in αB-crystallin increase the chaperone activity by preventing heat shock- and reduction-induced aggregation of target proteins, although for some substrates the chaperone activity might be decreased depending on the nature and concentration of the substrates [12–15].

Previously, we and others have found that a small subset of the αB-crystallin population can be found in the nucleus, where it predominantly is localized in nuclear speckles [16–19],. These nuclear speckles, also known as interchromatin granule clusters (IGCs), are thought to be sites for storage and recycling of splicing factors [20]. The subnuclear localization of αB-crystallin is modulated by differential phosphorylation of Ser-45 and Ser-59, as demonstrated by the substitution of these serine residues by negatively charged aspartate residues to mimic phosphorylation, or by non-phosphorylatable alanine residues, as well as by immunocytochemical staining with antibodies directed to endogenously phosphorylated αB-crystallin [18]. In interphase cells, phosphorylation at Ser-59 is required for nuclear import, whilst phosphorylation at Ser-45 is crucial for nuclear speckle localization [18]. Furthermore, nuclear import of αB-crystallin might be facilitated by the Survival Motor Neuron (SMN) complex, as suggested by the interaction and co-localization of (pseudo) phosphorylated αB-crystallin with a subunit of this complex [18]. The SMN complex is involved in the biogenesis and nuclear import of small nuclear ribonucleoparticles (snRNPs) [21,22] and contains, in addition to SMN, at least seven other proteins, named Gemin2-8 [23].

The highly regulated nuclear import of αB-crystallin and its specific subnuclear localization suggest that this protein has a nuclear function in addition to its activities in the cytoplasm. Here we show that upon heat shock pseudophosphorylated αB-crystallin moves from the nuclear speckles to the nucleoplasm, allowing protection of a broad range of nuclear proteins, and we identified the SMN complex as an important factor that facilitates the nuclear import of αB-crystallin.

## Materials and Methods

### Cell culture, plasmids, transfections and two-hybrid screen

HeLa cells were grown at 37°C in Dulbecco’s modified Eagle’s medium (DMEM; Invitrogen) supplemented with 10% fetal calf serum (FCS; PAA laboratories), 100 units/ml penicillin and 200 µg/ml streptomycin, in the presence of 5% CO_2_ in a humidified 37^°^C incubator. The doxycycline-inducible T-REx™-HeLa-pcDNA4-αB-STD cell line [24] was kept in Eagle’s minimal essential medium (EMEM, Lonza) supplemented with Glutamax^tm^ (Invitrogen), 10% fetal calf serum (Gibco-BRL), 5 µg/ml Blasticidin (Invitrogen) and 200 µg/ml Zeocin (Invitrogen). The DNA fragments encoding the sequence of αB-crystallin wild-type, STD (Ser-19, Ser-45 and Ser-59 replaced by Asp) and STA (Ser-19, Ser-45 and Ser-59 replaced by Ala) have been described elsewhere [24,25]. The nuclear luciferase DNA, present in the pEGFP-N2 vector from Clontech has been described by Nollen and coworkers [26]. A β-galactosidase expression vector was used to measure the transfection efficiency in the nuclear luciferase assay [27]. Transfections of HeLa cells with the plasmids were performed by lipofection using the FuGENE^tm^ 6 system (Roche Molecular Biochemicals), as described by the manufacturer. The yeast two-hybrid screen was performed with αB-crystallin-LexA fusion protein (bait) to select for proteins (preys) able to interact with αB-crystallin by screening a cDNA library from HeLa cells as has been described before [28].

### Heat shock assay

HeLa cells (1 x 10^6^) were seeded on coverslips (18 x 18 mm^2^) one day prior to transfection with 1 µg DNA. Two days after transfection cells were untreated or heat shocked for 45 minutes at 45^o^C and recovered at 37^o^C for the indicated time. The cells were fixed in 3% paraformaldehyde for 15 minutes and permeabilized for 10 minutes in 0.2% Triton X-100 in phosphate-buffered saline (PBS). The cells were subsequently incubated with undiluted monoclonal antibody to αB-crystallin (RIKEN, Cell Bank) and a 1:20 dilution of TRITC-conjugated rabbit anti-mouse antibody (DAKO Corp.). All images were obtained by confocal laser scanning microscopy (BIO-RAD MRC1024).

### Nuclear luciferase refolding assay

HeLa cells (1 x 10^5^) were transfected with 0.2 µg of the transfection control vector expressing β-galactosidase, 0.2 µg pN-luc-EGFP vector coding for nuclear luciferase and 0.6 µg of the pIRES vector either empty or with an αB-crystallin isoform. Three days after transfection, cells were supplemented with 100 µg/ml cycloheximide and 20 mM 3-(N-morpholino)propanesulfonic acid (MOPS) pH 7.4, 1 hour prior to heat shock. Cells were incubated at 45°C for 45 minutes, followed by recovery at 37°C in the presence of 5% CO_2_. Cells were washed 3 times with PBS and lysed by scraping the cells in 100 µl nuclear luciferase lysis buffer (25 mM Tris/H_3_PO_4_, pH 7.8, 10 mM MgCl_2_, 1% Triton X-100, 15% glycerol and 1 mM EDTA) per 10^6^ cells. Luciferase activity was measured by adding 50 µl of luciferase reagent (Promega) to 10 µl of lysate and subsequent analysis on a Lumat LB 9507 luminometer. β-Galactosidase activity was determined by first incubating 10 µl lysate for 30 minutes with 100 µl of β-galactosidase reagent buffer (1:100 Galacton (Tropix) in 100 mM phosphate buffer pH 8.1, 5 mM MgCl_2_) and then adding 150 µl light emission accelerator (Tropix) for the analysis on a Lumat LB 9507 luminometer.

### Co-Immunoprecipitations

HeLa cells (1 x 10^6^) were transfected with 1 µg pIRES vector either empty or with an αB-crystallin isoform. Cells were harvested by trypsinization and washed once with DMEM containing 10% fetal calf serum and twice with cold PBS, and suspended in 1 ml lysis buffer (50 mM Tris-HCl pH 7.5, 100 mM NaCl, and 0.5% Nonidet P-40) at 4°C. Cell lysates were cleared by centrifugation at 16,000 g for 30 minutes at 4°C and subsequently incubated with protein G-agarose beads (Roche Molecular Biochemicals) coupled with monoclonal antibody to αB-crystallin (RIKEN, Cell Bank). After incubation at 4°C, beads were washed three times with buffer (50 mM Tris-HCl pH 7.5, 100 mM NaCl, and 0.05% Nonidet P-40) and boiled in sample buffer (1% SDS, 0.063 M Tris-HCl pH 6.8, 10% glycerol, 0.01% β-mercaptoethanol, 0.02% bromophenol blue) and analyzed by western blotting.

### In vitro import assay [29]

HeLa cells grown on coverslips to 80-90% confluence were washed twice with PBS and once with P-buffer (50 mM HEPES/KOH pH 7.5, 50 mM KAc, 8 mM MgAc_2_, 2 mM EGTA, 1 mM DTT, protease inhibitors cocktail (Roche)). The cells were permeabilized for 5 minutes at room temperature with 40 mg/ml digitonin (Sigma) in T-buffer (20 mM HEPES/KOH pH 7.5, 50 mM KAc, 4 mM MgAc_2_, 1mM DTT, protease inhibitors cocktail (Roche) and subsequently washed three times with T-buffer. The permeabilized cells were incubated with 50 µl reaction buffer (T-buffer containing 1 mM ATP, 0.2 mM GTP, 5 mM phosphocreatine, 0.2 mg/ml creatine phosphokinase, 10 mg/ml BSA, 0.25 M sucrose), which was mixed with 5 µl reticulocyte lysate (Promega) and 400 µg purified recombinant αB-crystallin [28], labeled with FITC (Fluorescein isothiocyanate, Pierce) according to the protocol described by the manufacturer, and incubated for 60 minutes at room temperature. After incubation, the cells were washed three times with T-buffer and fixed with 3% paraformaldehyde and mounted on glass slides with Fluorescent Mounting Medium (Dakocytomation). Images of the cells were obtained by confocal laser scanning microscopy (Bio-Rad MRC1024). Immunodepletion reticulocyte lysate was performed by incubating 20 µl lysate for 30 minutes with protein G beads alone or with protein G beads coupled with Gemin3 or SMN protein antibodies, after which the beads were removed by centrifugation. Nuclear import was reconstituted by adding 50 ng SMN complex, which was purified from HeLa cells stably overexpressing Flag-tagged SMN [30] (see Figure S2).

### siRNA transfection assay

T-REx™-HeLa-pcDNA4-αB-STD cell line was seeded on coverslips (10 mm^2^) in the absence of antibiotics. At 40% confluency, cells were transfected with 0.1 ng siRNA per coverslip (n=2 per sample) using Lipofectamine^tm^ 2000 Reagent according to manufacturers’ protocol (Invitrogen). Six different siRNA’s were used: two negative control siRNAs: si-Luciferase (CGUACGCGGAAUACUUCGAdTdT), si-Pop1 (GAAUUUAACCGUAGACAAAdTdT) [31], two Gemin2 specific siRNA’s: si-Gemin2.1 (CCCAACACUUCAAUGGCAAdTdT), si-Gemin2.2 (CUGGAAUAGAUUAUGUACAdTdT) and two Gemin3 specific siRNAs: si-Gemin3.1 (GAGGAGUACUGGAGAGCUUdTdT), si-Gemin3.2 (GCAAAGGAAAUAAGUCAUAdTdT). The medium was refreshed 5 hours after transfection and 24 hours after transfection 1 µg/µl doxycycline (Invitrogen) was added. After 16 hours culturing with doxycycline, cells were washed twice with PBS and fixed for 20 minutes at RT in 2% paraformaldehyde (Merck) in PBS and subsequently washed with PBS-G (PBS supplemented with 1.5 g/l glycine (MP Biomedicals)). Cells were permeabilized by 10 minutes incubation at RT with 0.1% Triton X100 (USB) in PBS-G. Slides were incubated with monoclonal antibody to αB-crystallin (RIKEN, Cell Bank) for 1 hour at 37^o^C, 10-fold diluted in PBS-GT, (PBS-G, containing 0.05% Tween-20 (Fluka)), washed three times with PBS-G and incubated with goat-α-mouse-Alexa Fluor 488 (LI-COR) for 1 hour at 37^o^C, 1:150 diluted in PBS-GT. Coverslips were washed three times with PBS-G and mounted using MOWIOL (Calbiochem). Analysis was performed with a fluorescence microscope (Axioskop, Zeiss). Per glass slide, 3 fields were selected randomly and per siRNA an average of 110 cells were counted. Speckle positive and negative nuclei were scored by eye by two independent evaluators. Statistical analysis was performed using One-way ANOVA and Tukey’s Multiple Comparison Test.

To check for transfection efficiency, cells were transfected using 1.25 ng siRNA/T25 flask. The cells were harvest by trypsinization, washed two times with PBS and denatured by 5 minutes heating in 40 µl sample buffer and analyzed by western blotting.

### Western blotting

Protein samples were separated by electrophoresis on a 12.5% SDS-polyacrylamide gel and transferred to nitrocellulose membranes (Protran) or Hybond-P membranes (Amersham Biosciences). The membranes were blocked with 5% non-fat dried milk (Campina) + 0.05% Tween-20 (Merck) in PBS for an hour. The membranes were incubated O/N in 2% non-fat dried milk in PBS containing monoclonal mouse-anti-human-αB-crystallin (RIKEN), mouse-anti-human-gamma-tubulin (Sigma-Aldrich), mouse-anti-human-gemin2 (E6, Santa Cruz Biotechnology), mouse-anti-human-gemin3 (12H12, Santa Cruz Biotechnology), rabbit-anti-human-αB-crystallin [32], rabbit-anti-human-SMN (Santa Cruz Biotechnology) or rabbit-anti-human-Skp1 (Neomarkers). After washing three times ten minutes with PBS complemented with 0.1% Tween-20, bGAAUUUAACCGUAGACAAAdTdT lots were incubated for one hour with goat-anti-mouse IRDye^®^ 800CW (LI-COR) or horseradish peroxidase conjugated goat-anti-mouse secondary antibodies or swine-anti-rabbit secondary antibodies (DAKO Corp.) to allow visualization of the bound antibodies with the Odyssey scanner (LI-COR) or by enhanced chemoluminescence (Pierce Chemical Co.).

## Results

### Speckle-localized pseudophosphorylated αB-crystallin serves as a chaperone depot

**Figure 1:**
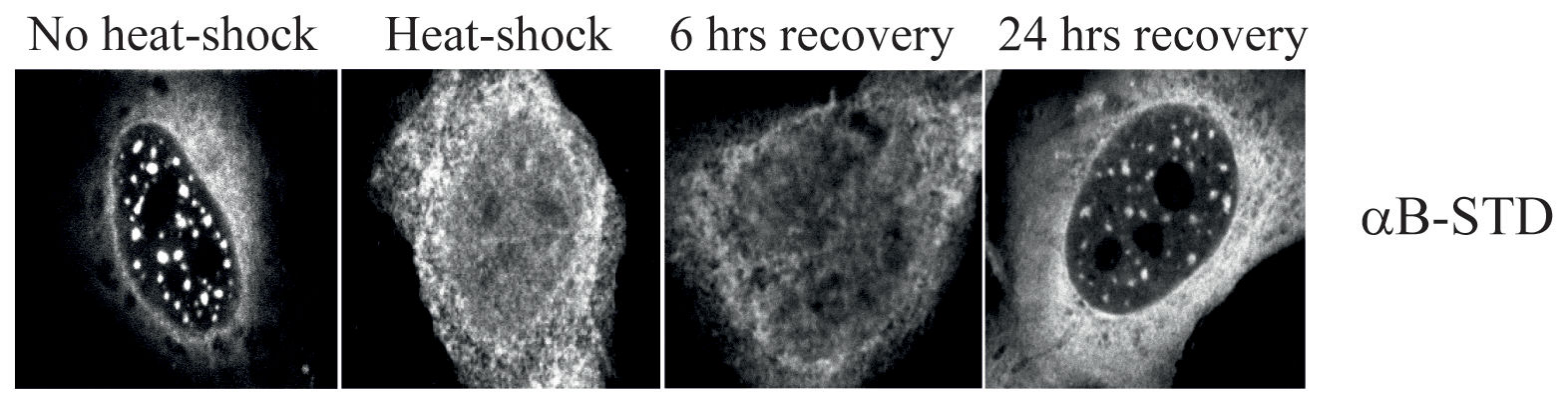
Relocalization of nuclear pseudophosphorylated αB-crystallin in response to heat-shock. Transiently transfected HeLa cells expressing αB-STD were mock-treated or subjected to heat-shock and allowed to recover for 6 or 24 hours. The localization of αB-STD was visualized by immunofluorescence, using a monoclonal antibody against αB-crystallin.

Under normal physiological conditions αB-crystallin is mainly present in the cytoplasm, but upon phosphorylation at Ser-59 and Ser-45 accumulates in nuclear speckles [18]. To investigate whether αB-crystallin remains in the nuclear speckles upon heat shock, the pseudophosphorylated αB-crystallin mutant αB-STD was used. This mutant, besides being present in the cytoplasm, abundantly accumulates in the nuclear speckles [18]. HeLa cells transfected with pIRES αB-STD vector were subjected to a heat shock (45 min) and subsequently allowed to recover for 0, 6 and 24 hours at 37°C. As expected, nuclear αB-STD of non-heat shocked cells was found predominantly in speckles. However, heat-shocked cells examined directly or 6 hours after the heat shock showed diffuse nuclear staining (Figure 1). Speckle localization was restored 24 hours after heat shock. These results suggest that during heat stress nuclear αB-crystallin exerts its chaperone function in the nucleoplasm.

### Pseudophosphorylated *αB*-crystallin is a nuclear chaperone

**Figure 2:**
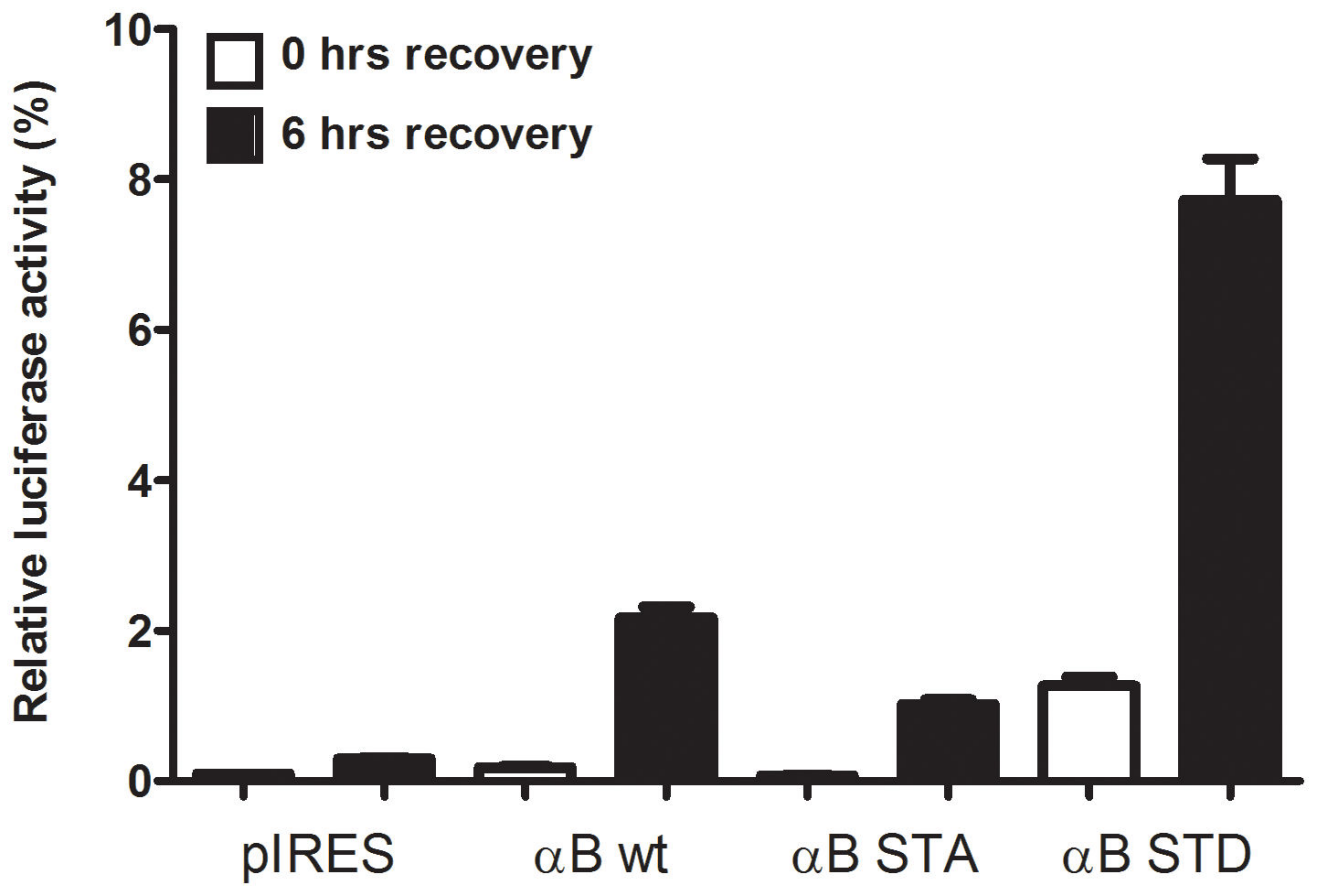
Pseudophosphorylated αB-crystallin enhances refolding of heat-inactivated nuclear luciferase. Transiently transfected HeLa cells expressing nuclear luciferase were heated (45 min at 45^o^C) and subsequently lysed directly or 6 hours thereafter. Cycloheximide was added just prior to heating to prevent de novo luciferase synthesis. Recovery of luciferase activity is indicated for cells cotransfected with empty pIRES vector (pIRES), or constructs encoding wild-type (WT), non-phosphorylatable αB-crystallin (αB-STA) or pseudophosphorylated αB-crystallin (αB-STD). The luciferase activity was determined and compared with the initial luciferase activity before heat shock. All transfections were corrected for the transfection efficiency as measured by the β-galactosidase activity resulting from a cotransfected β-galactosidase expression vector. The results represent the mean values of 6 independent experiments; error bars indicate the standard deviation. A western blot illustrating the expression of αB-crystallin is shown in Figure S1.

To determine whether αB-crystallin can assist in the refolding of proteins present in the nucleus we used a nuclear targeted luciferase-EGFP fusion protein (N-luc-EGFP) [26] as a heat-sensitive reporter. This reporter protein shows a diffuse nuclear staining in non-stressed cells. HeLa cells were transfected with the empty pIRES vector or with pIRES constructs coding for αB-crystallin or mutants thereof, together with the nuclear luciferase construct. In every transfection a β-galactosidase construct was included to be able to correct for variations in transfection efficiency. After three days the cells were exposed to a heat shock to denature the luciferase. The luciferase activity was measured before, directly after and six hours after heat shock. During the recovery period the translation inhibitor cycloheximide was present to prevent *de novo* synthesis of luciferase. As can be seen in Figure 2, directly after heat shock the activity of luciferase was strongly reduced in cells transfected with pIRES, wild-type or non-phosphorylatable αB-crystallin (αB-STA) and somewhat less pronounced in the cells transfected with αB-STD. Six hours after heat shock a significant recovery of luciferase activity was observed in the αB-STD transfected cells (8.7%), whereas the pIRES, wild-type or αB-STA transfected cells showed almost no or much less recovery of luciferase activity (0.3, 2.2 and 1.0%, respectively). These data demonstrate that pseudophosphorylated αB-crystallin participates in nuclear chaperoning activity during and directly after heat shock.

### Pseudophosphorylated αB-crystallin interacts with Gemin3

**Figure 3:**
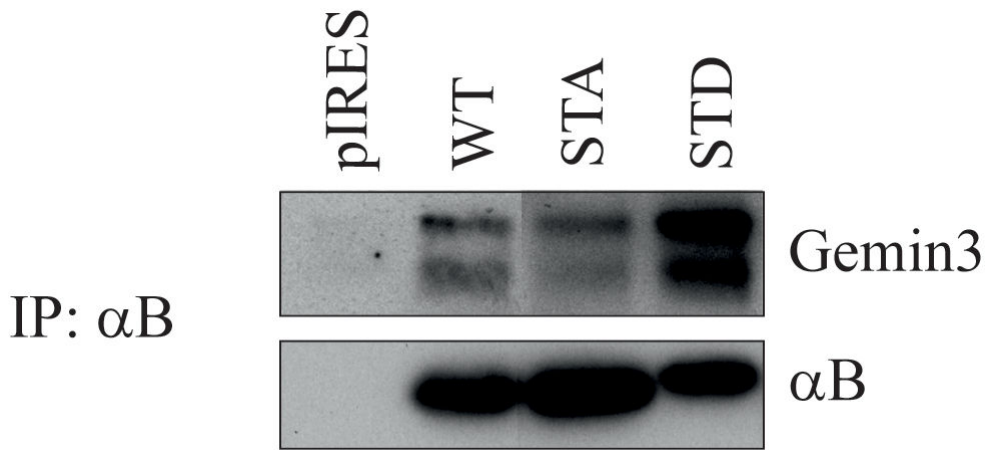
Co-immunoprecipitation of Gemin3 with αB-crystallin. Extracts of HeLa cells, transfected with pIRES as a control or pIRES constructs coding for wild-type (WT), non-phosphorylatable αB-crystallin (αB-STA) or pseudophosphorylated αB-crystallin (αB-STD) were subjected to immunoprecipitation with a monoclonal antibody against αB-crystallin. The immunoprecipitates were analyzed by immunoblotting using a mouse monoclonal antibody against Gemin3 and rabbit polyclonal antibodies to αB-crystallin. Note that Gemin3 shows two bands, of which the lower band is likely a degradation product.

Pseudophosphorylated αB-crystallin does not contain a canonical nuclear localization signal and likely enters the nucleus via an alternative route by binding to a specific adaptor complex. In a yeast two-hybrid system we found that wild-type αB-crystallin is able to interact with the C-terminal 246 residues of Gemin3 (data not shown). Gemin3 is a subunit of the SMN complex [33] and we have previously shown that αB-STD, but not the non-phosphorylatable αB-STA, can interact with the SMN protein, another subunit of the same complex [18]. The SMN complex facilitates the assembly of snRNPs and their import into the nucleus [22], to allow partial accumulation in nuclear speckles. Since αB-STD also localizes in these speckles, we hypothesized that the interaction with this complex facilitates the nuclear accumulation of αB-STD [18]. To confirm the interaction of αB-crystallin with Gemin3, a co-immunoprecipitation experiment was performed, using lysates of HeLa cells cotransfected with different αB-crystallin expression constructs. The wild-type and mutant αB-crystallin were efficiently precipitated with anti-αB-crystallin antibody (Figure 3 lower panel). Gemin3 co-immunoprecipitated with wild-type αB-crystallin (Figure 3 upper panel), which confirms the interaction observed in the two-hybrid system. The efficiency of the coprecipitation of Gemin3 with αB-STA was similar as that observed for the wild-type αB-crystallin, but appeared to be much higher with αB-STD. These results show that αB-crystallin is able to interact with Gemin3 and suggest that the affinity of this interaction is increased by the phosphorylation of αB-crystallin.

### Nuclear localization of pseudophosphorylated αB-crystallin is facilitated by Gemin3 and Gemin2

**Figure 4:**
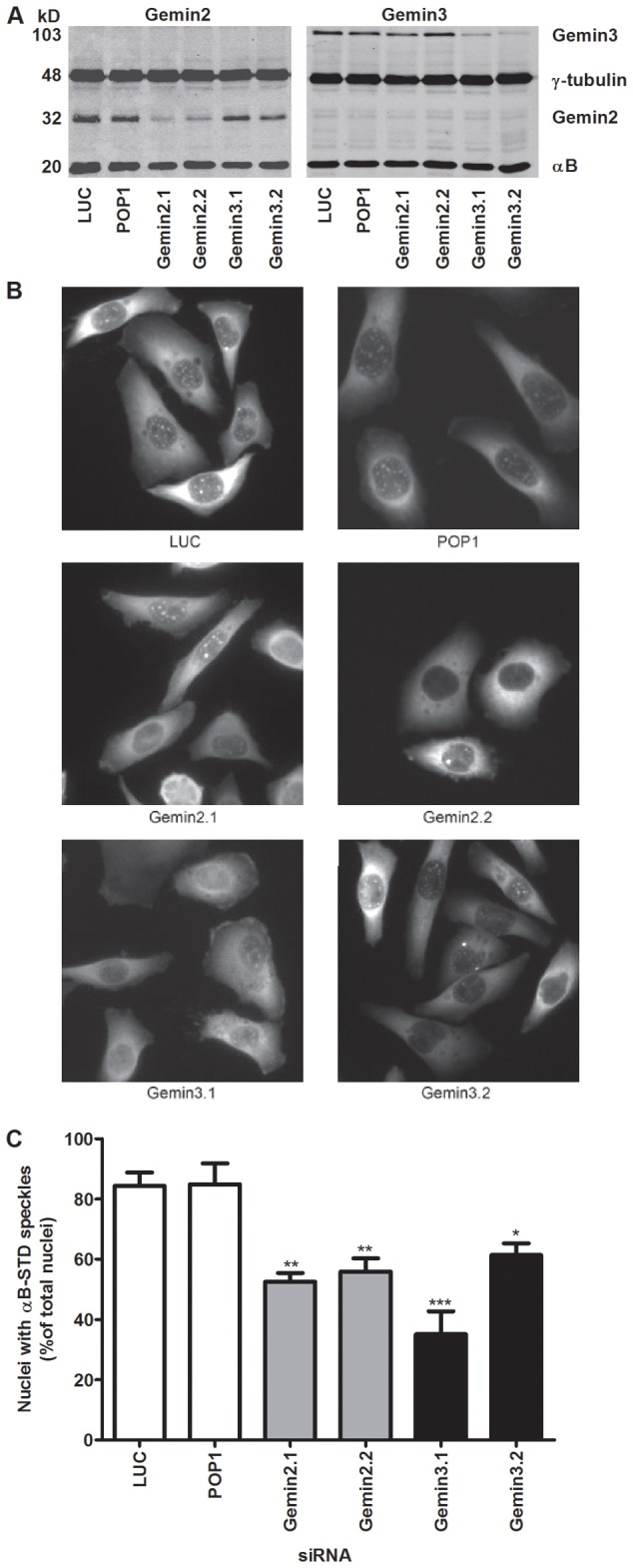
The effect of knockdown of Gemin3 and Gemin2 on the nuclear import of pseudophosphorylated αB-STD. Doxycycline-inducible T-REx™-HeLa-αB-STD cells were treated with two different Gemin3 siRNAs, two different Gemin2 siRNAs and two negative control siRNAs (LUC and POP1). After 24 hours 1 µg/µl doxycycline was added to induce αB-STD expression and 16 hours later the cells were analyzed. (A) Knockdown of Gemin3 and Gemin2 was assessed by western blotting analysis. αB-crystallin and γ-tubulin were used as loading controls. (B) T-REx™-HeLa-αB-STD cells treated with the different siRNAs were stained for αB-crystallin. (C) The percentages of the siRNA-treated cells containing αB-crystallin-positive speckles in the nucleus were determined and are shown in the graph. Statistical analysis was performed using One-way ANOVA and Tukey’s Multiple Comparison Test. *** P<0.001, ** 0.001<P<0.01, * 0.01<P<0.05.

To investigate whether nuclear speckle localization of αB-STD is Gemin3-dependent, stably transfected HeLa αB-STD cells were used with doxycycline-inducible expression of αB-STD. The expression of αB-STD was induced 24 hours after transfection with siRNAs directed against Gemin3. αB-crystallin was visualized by immunostaining and the number of cells containing αB-crystallin-positive nuclear speckles was determined. As can be seen in Figure 4B and 4C, 84% and 85% of cells that were treated with unrelated control siRNAs (Luciferase and POP1) showed nuclear speckle staining. This was comparable to cells that were not transfected with siRNA, indicating that siRNA treatment did not influence the presence of αB-STD in the nuclei (results not shown). Two distinct siRNAs (Gemin3.1 and Gemin3.2) resulted in down-regulation of Gemin3 expression, as demonstrated by western blotting (Figure 4A). Knockdown of Gemin3 with these two siRNAs resulted in a significant reduction of cells containing αB-STD positive speckles (Figure 4B and 4C, 35% and 61%, respectively). To confirm the involvement of the SMN complex in αB-STD import [34], also Gemin2 expression was down-regulated with two different siRNAs (Figure 4A). Gemin2 depletion also resulted in decreased numbers of cells containing αB-STD positive speckles (Figure 4B and 4C, 52% and 56% respectively). These results strongly suggest that the SMN complex is involved in nuclear speckle localization of pseudophosphorylated αB-crystallin.

### Nuclear import of pseudophosphorylated αB-crystallin is facilitated by the SMN complex

**Figure 5:**
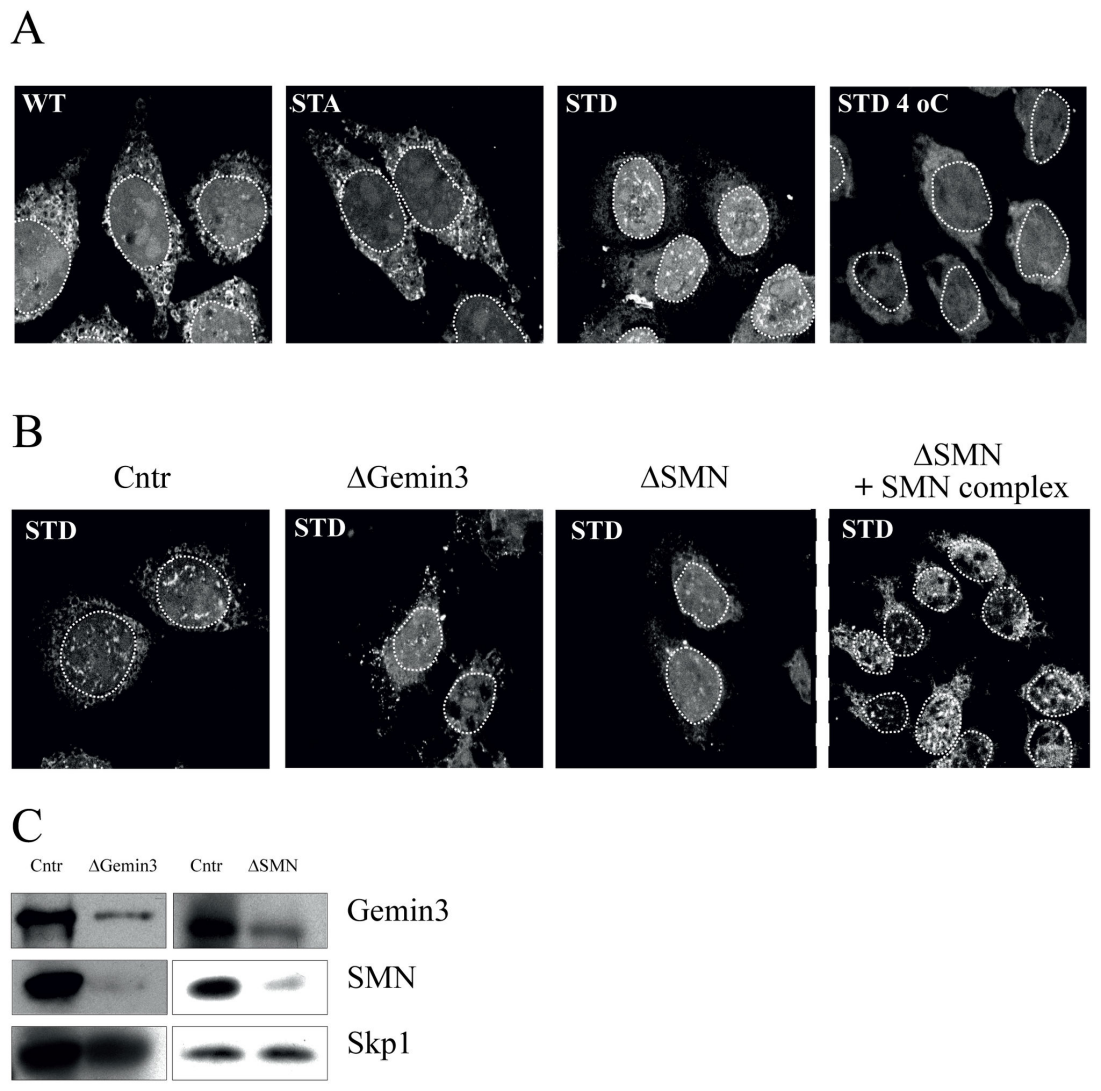
*In vitro* nuclear import of pseudophosphorylated αB-crystallin is dependent on the SMN complex. *In vitro* nuclear import assays were conducted by incubating digitonin-permeabilized HeLa cells for 1 hour at room temperature with reticulocyte lysates containing ATP and GTP. (A) Import reactions were performed with FITC-labeled recombinant wild-type αB-crystallin (WT), non-phosphorylatable αB-crystallin (αB-STA) and pseudophosphorylated αB-crystallin (αB-STD). As a negative control the import assay with αB-STD was conducted at 4°C (STD 4°C). (B) Import reactions of αB-STD with rabbit reticulocyte lysates immunodepleted with anti-Gemin3 antibodies (ΔGemin3), anti-SMN antibodies (ΔSMN) and control-depleted (Cntr). The impaired import of αB-STD with SMN-depleted reticulocyte lysate could be rescued by addition of purified SMN complex (ΔSMN+ SMN complex). (C) The immunodepleted reticulocyte lysates were analyzed by western blot to assess depletion. Skp1 was used as a loading control. Dotted circles indicate the position of the nuclei.

To obtain additional evidence for the involvement of complete SMN complex in nuclear import of αB-STD, an *in vitro* nuclear import system with digitonin-permeabilized HeLa cells was used [35]. Digitonin permeabilizes the cell membrane, whilst the nuclear membrane remains intact, allowing to reconstitute nuclear import by reticulocyte lysate containing ATP and GTP. First, we tested whether fluorescently labeled recombinant αB-STD was indeed imported into the nucleus in this system. The results showed that αB-STD was not only imported into the nucleus, but was also targeted to nuclear speckles (Figure 5A), which was confirmed by the colocalization with the nuclear speckle marker SC35 (data not shown). Thus, the *in vitro* localization of αB-STD is similar to that observed in transiently transfected cell lines [24]. The *in vitro* nuclear import of αB-STD is an active process, as shown by the inhibition of import at 4°C (Figure 5A). In contrast to αB-STD, fluorescently labeled wild-type and non-phosphorylatable αB-crystallin (αB-STA) only showed background staining in the nucleus, indicating that these proteins were not imported into the nucleus, which agrees with previous *in situ* results [24].

To test whether import of pseudophosphorylated αB-crystallin depends on the SMN complex we immunodepleted reticulocyte lysates from this complex using antibodies directed to SMN and Gemin3. As can be seen in Figure 5C, the Gemin3- and SMN-depleted lysates indeed contained strongly reduced levels of Gemin3 and SMN. Importantly, Gemin3 depletion resulted in efficient co-depletion of SMN, and vice versa, consistent with the removal of the complete SMN complex by this procedure. The SMN-depleted lysates resulted in a significant reduction of the accumulation of αB-STD in nuclear speckles (Figure 5B). To confirm that these effects were due to depletion of the SMN complex, purified SMN complex was added to the SMN-depleted reticulocyte lysate. The addition of the SMN complex indeed rescued the import and speckle localization of αB-STD (Figure 5B). These results show that nuclear import of pseudophosphorylated αB-crystallin is facilitated by the SMN complex. 

## Discussion

Differential phosphorylation of αB-crystallin is required for nuclear localization [18] and occurs depending on cell cycle and cellular status [10,11]. Nuclear speckle accumulation of αB-crystallin can be enhanced by mimicking phosphorylation at three phosphorylation sites [18]. Here we show that upon heat shock, the pseudophosphorylated αB-crystallin does not reside in nuclear speckles, but is distributed over the nucleoplasm and re-accumulates in the speckles after release from stress. This is in concurrence with findings of Van den IJssel and coworkers, who showed that the speckle-localized endogenous αB-crystallin in U373 cells significantly diminished after heat shock [16]. Nucleoplasmic αB-crystallin is able to protect nuclear proteins, as shown by the ability of pseudophosphorylated αB-crystallin to enhance the refolding of heat-shock-denatured nuclear luciferase. These results suggest that nuclear speckle-localized αB-crystallin, besides acting as a molecular chaperone for nuclear speckle proteins under non-stressed conditions, serves as a depot to allow a rapid increase of αB-crystallin in the nucleoplasm to respond to heat stress. Nuclear αB-crystallin has been shown to help to prevent methylglyoxal-induced apoptosis in retinal pigment epithelial cells, indicating that nuclear αB-crystallin also has a protective role under other stress conditions [36].

αB-crystallin does not play an active role in the refolding of partially unfolded proteins, though it has been proposed to keep substrate proteins in a folding-competent state, thereby facilitating ATP-dependent refolding by other molecular chaperones, such as Hsp70 [9]. During heat stress, importin β-mediated nucleocytoplasmic trafficking is strongly reduced, thereby preventing the active import of cytoplasmic proteins into the nucleus. In contrast to most cytoplasmic proteins, nuclear import of Hsp70 is upregulated during heat shock [37], which is mediated by a novel nuclear import carrier, named Hikeshi [38,39]. The nuclear Hsp70 might subsequently act in concert with αB-crystallin to restore heat shock-induced damage inside the nucleus.

αB-crystallin forms polydisperse complexes containing 10 to 40 subunits. Pseudophosphorylation reduces the size of the oligomeric complex of αB-crystallin [12,14,40], but these complexes are still too large to allow passive diffusion into the nucleus, as shown by the lack of nuclear import of αB-STD at 4°C (Figure 5A). Because of the absence of a recognizable nuclear localization signal sequence [19] the nuclear import of αB-crystallin is expected to be mediated by an alternative import carrier. A clue for such a carrier came from yeast-two-hybrid experiments, which demonstrated that αB-crystallin interacts with the C-terminal 246 residues of Gemin3. Although the non-phosphorylated αB-crystallin can interact with Gemin3, in co-immunoprecipitation experiments, Gemin3 was more efficiently pulled down by pseudophosphorylated αB-crystallin, suggesting that hyperphosphorylation increases the affinity for Gemin3. Pseudophosphorylation reduces the interaction between the αB-crystallin subunits [40], which might facilitate the binding to Gemin3. Consistent with the involvement of Gemin3, as a subunit of the SMN complex, in subcellular transport processes [34], Gemin3 appeared to mediate nuclear import of pseudophosphorylated αB-crystallin. This activity of Gemin3 is probably related to its association with the SMN complex, as demonstrated by additional experiments in which the levels of Gemin2 and SMN were reduced. These data strongly suggest that the SMN complex is responsible for nuclear import of αB-STD. SMN is able to directly interact with importin β, as shown in a GST-pull down assay [41], which might facilitate the interaction with the nuclear pore complex to stimulate the nuclear import of the complex [42]. Since SMN colocalizes with phosphorylated αB-crystallin in the nuclear speckles [18], it is likely that this complex is also involved in the accumulation of nuclear αB-crystallin in speckles.

Besides αB-crystallin, the C-terminus of Gemin3 is also able to interact with another member of the small heat shock protein family, HspB8 [43]. However, so far no evidence for the accumulation of HspB8 in nuclear speckles has been obtained [19].

Two other small heat shock proteins have been described to localize to nuclear speckles, HspB1 and HspB7. V5-tagged HspB7 constitutively localizes to nuclear speckles, indicating that posttranslational modifications are not involved or are constitutively present. The nuclear speckle localization signal is situated in the N-terminus of HspB7, but it is not known whether this part is able to interact with Gemin3. Speckle-localized HspB7 does not enhance refolding of heat-denatured nuclear firefly luciferase, suggesting that the association with the nuclear speckles is not related to protein refolding [19].

The accumulation of HspB1 in nuclear speckles depends on more than one signal. Upon stress HspB1 is phosphorylated by the mitogen-activated protein kinase (MAPK) pathway. Phosphorylation of HspB1 has been shown to be necessary for recruitment to speckles, but by itself is not sufficient to support nuclear import [44]. Therefore, the phosphorylation of HspB1 may have a similar function as the Ser-45 phosphorylation of αB-crystallin [18]. Heat stress induces entrance of HspB1 into the nucleus, which suggests the involvement of a heat shock activated nuclear import factor. αB-STD is able to recruit endogenous HspB1 to nuclear speckles without an additional heat shock (unpublished data), indicating that αB-crystallin is capable to stimulate nuclear import of HspB1. Since heat stress stimulates nuclear accumulation of αB-crystallin [45], it is possible that αB-crystallin in part is responsible for the heat shock-induced nuclear accumulation of HspB1. Nuclear HspB1 has been shown to contribute to increased chaperone capacity in the nucleus under stress conditions, which, similar to the activity of αB-crystallin, seems to be localized outside the nuclear speckles [46].

In summary, our data suggest that phosphorylated αB-crystallin present in nuclear speckles of unstressed cells temporarily moves to the nucleoplasm upon heat shock, providing a rapid increase in chaperone capacity to bind unfolded proteins. The import of phosphorylated αB-crystallin into the nucleus is facilitated by the SMN complex via the interaction with Gemin3. Further investigations are needed to shed more light on the functional activities of αB-crystallin and other small heat-shock proteins in the nucleus, both under normal and under stress conditions.

## Supporting Information

Figure S1 **αB-crystallin expression.** Expression of wild-type αB-crystallin (WT), non-phosphorylatable αB-crystallin (αB-STA) or pseudophosphorylated αB-crystallin (αB-STD) in transiently transfected HeLa cells. αB-crystallin expression was analyzed by immunoblotting using a monoclonal antibody.
(TIF)

Figure S2 **Composition of purified SMN complex.** The purified SMN complex was separated by SDS-polyacrylamide gel electrophoresis, followed by silver staining. The SMN complex was purified form HeLa cells stably expressing Flag-tagged SMN protein using anti-FLAG-tag antibodies. The complex was eluted with an excess of Flag-tag peptide. The control lane (Cntr) contains proteins isolated in parallel by the same procedure from control HeLa cells.
(TIF)
