# Supplementary figures and images for: Pseudophosphorylated αB-Crystallin Is a Nuclear Chaperone Imported into the Nucleus with Help of the SMN Complex
Source: PLoS One. 2013 Sep 4;8(9):e73489. doi: 10.1371/journal.pone.0073489 (PMC3762725; doi:10.1371/journal.pone.0073489)

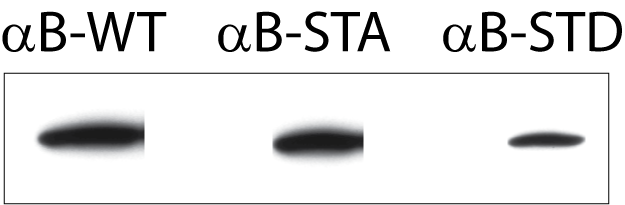

Supplement: Figure S1 — αB-crystallin expression. Expression of wild-type αB-crystallin (WT), non-phosphorylatable αB-crystallin (αB-STA) or pseudophosphorylated αB-crystallin (αB-STD) in transiently transfected HeLa cells. αB-crystallin expression was analyzed by immunoblotting using a monoclonal antibody. (TIF) [file pone.0073489.s001.tif]

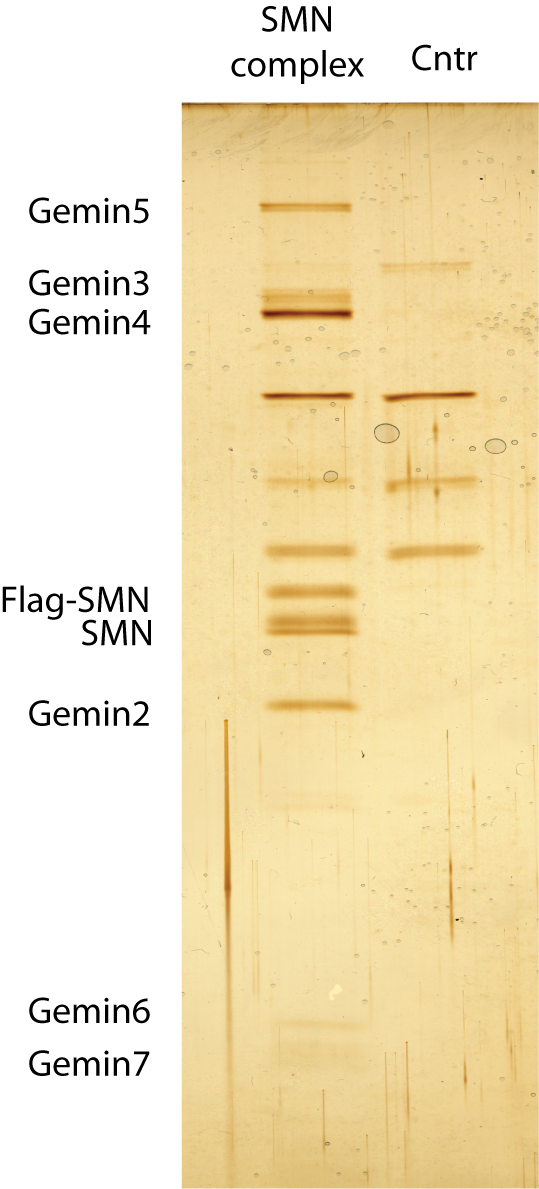

Supplement: Figure S2 — Composition of purified SMN complex. The purified SMN complex was separated by SDS-polyacrylamide gel electrophoresis, followed by silver staining. The SMN complex was purified form HeLa cells stably expressing Flag-tagged SMN protein using anti-FLAG-tag antibodies. The complex was eluted with an excess of Flag-tag peptide. The control lane (Cntr) contains proteins isolated in parallel by the same procedure from control HeLa cells. (TIF) [file pone.0073489.s002.tif]
